# Toll-like receptor 9 and 4 gene polymorphisms in susceptibility and severity of malaria: a meta-analysis of genetic association studies

**DOI:** 10.1186/s12936-021-03836-6

**Published:** 2021-07-03

**Authors:** Cho Naing, Siew Tung Wong, Htar Htar Aung

**Affiliations:** 1 Institute for Research, Development and Innovation (IRDI), International Medical University, 5700 Kuala Lumpur, Malaysia; 2 Faculty of Tropical Heath and Medicine, James Cook University, Queensland, Australia; 3 School of Medicine, International Medical University, Kuala Lumpur, Malaysia

**Keywords:** Toll-like receptor, Polymorphisms, Severity, Susceptibility, Malaria, Meta-analysis

## Abstract

**Background:**

Malaria is still a major public health problem in sub-Saharan Africa and South-east Asia. The clinical presentations of malaria infection vary from a mild febrile illness to life-threatening severe malaria. Toll like receptors (TLRs) are postulated to be involved in the innate immune responses to malaria. Individual studies showed inconclusive findings. This study aimed to assess the role of TLR4 (D299G, T399I) and TLR9 (T1237C, T1486C**)** in severity or susceptibility of malaria by meta-analysis of data from eligible studies.

**Methods:**

Relevant case–control studies that assessed the association between TLR 4/9 and malaria either in susceptibility or progression were searched in health-related electronic databases. Quality of included studies was evaluated with Newcastle–Ottawa scale. Pooled analyses for specific genetic polymorphisms were done under five genetic models. Stratified analysis was done by age and geographical region (Asian countries vs non-Asian countries).

**Results:**

Eleven studies (2716 cases and 2376 controls) from nine endemic countries were identified. Five studies (45.4%) obtained high score in quality assessment. Overall, a significant association between TLR9 (T1486C) and severity of malaria is observed in allele model (OR: 1.26, 95% CI: 1.08–1.48, *I*^2^ = 0%) or homozygous model (OR: 1.55, 95% CI: 1.08–2.28, *I*^2^ = 0%). For TLR9 (T1237C), a significant association with severity of malaria is observed in in heterozygous model (OR:1.89, 95% CI: 1.11–3.22, *I*^2^ = 75%). On stratifications, TLR9 (T1486C) is only significantly associated with a subgroup of children of non-Asian countries under allele model (OR: 1.25, 95% CI: 1.02–1.38), while 1237 is with a subgroup of adults from Asian countries under heterozygous model (OR: 2.0, 95% CI: 1.09–3.64, *I*^2^ = 39%). Regarding the susceptibility to malaria, TLR9 (T1237C) is significantly associated only with the children group under recessive model (OR: 2.21, 95% CI: 1.06–4.57, *I*^2=^85%) and homozygous model (OR: 1.49, 95% CI: 1.09–2.0, *I*^2^ = 0%). For TLR4 (D299G, T399I), none is significantly associated with either severity of malaria or susceptibility to malaria under any genetic models.

**Conclusions:**

The findings suggest that TLR 9 (T1486C and T1237C) seems to influence the progression of malaria, under certain genetic models and in specific age group of people from specific geographical region. TLR 9 (T1237C) also plays a role in susceptibility to malaria under certain genetic models and only with children of non-Asian countries. To substantiate these, future well designed studies with larger samples across endemic countries are needed.

**Supplementary Information:**

The online version contains supplementary material available at 10.1186/s12936-021-03836-6.

## Background

Malaria is still a major public health problem in sub-Saharan Africa and south-eastern Asia, and *Plasmodium falciparum* infection is prevalent in most of the endemic country [1], albeit with enhanced control measures. The clinical presentations of malaria infection vary from a mild febrile illness to life-threatening severe anaemia, acidosis and end-organ failure, even among individuals with little or no acquired anti-malarial immunity [2]. In any population endemic for malaria, some people are presented with active infection that might/might not be fatal to them and some of these individuals are carriers of the disease, while many of them are normal [3, 4]. Such differences in the immune responses can be explained from the molecular aspect where the variation in the genetic constitution of an individual could result in either compromised or active immune response during a pathogenic infection [4].

The mechanisms of immunity to malaria are complex, but are believed to involve the innate and adaptive immune responses that restrict both the liver and blood stage of the parasites in the human host [5, 6]. The long coevolution between hosts and *Plasmodium* species allowed the parasite to develop diverse mechanisms to escape the hosts’ adaptive immune system. As such, innate immune receptors also have a crucial role in the control of the disease since they are the first line of parasite recognition [7].

It has been postulated that human host-immune response to malaria infection is triggered by the innate immune system that shapes adaptive immunity [8] and evidence suggests that Toll like receptors (TLRs) are involved in the innate immune responses to a variety of pathogens including Plasmodium [9–11]. TLR are a family of receptors that recognize patterns (pattern recognition receptors or PRRs). These receptors are important for sensing molecular patterns associated with pathogens (pathogen-associated molecular patterns or PAMPs) as well as associated with cell damage (damage-associated molecular patterns or DAMPs) [7]. Studies had reported that microbial infection initiates TLR responses, and its interaction between TLRs and PAMPs results in the induction of an array of antimicrobial immune responses [9] as well as the development of acquired immunity [10]. Since the first description of mammalian TLR in 1997, there is a considerable advancement in the work of elucidating the role of TLRs in human diseases through the research of innate immune response which involved in *vivo* and in vitro studies [4].

A published review reported the role of TLR in protection against tuberculosis in a population of Colombians [12]. An earlier review had addressed the role of TLR 4 and 9 in severity of malaria only, excluding susceptibility aspect [13]. Individual studies showed the role of TLR4 (D299G) or TLR 9 (T1237C, T1486C) genetic polymorphisms on the occurrence/progression of malaria [14], while other studies had reported differently [15]. To be comprehensive, it is valuable to synthesize evidence on both aspects (susceptibility and severity). Moreover, a surge of new studies after publication of a review in 2017 and there also are availability of data for other polymorphisms such as TLR4 (T399I). On the whole, the objective of current study was to assess the role of TLR4 (D299G, T399I) and TLR9 (T1237C, T1486C**)** in severity or susceptibility of malaria by meta-analysis of data from eligible studies. The analysis of genetic factor(s) may serve as a practical approach in identification of patients who are at high risk for a specific infection or for poor disease progression, leading to more aggressive treatment intervention [4].

## Methods

The present meta-analysis study adhered Preferred Reporting Items for Systematic Reviews and Meta-Analyses Protocols (PRISMA) statement [16] (Additional file 1).

### Search strategy

Relevant studies published in English between 1995 and September 2020 were searched in electronic databases of PubMed, MEDLINE, EMBASE, Web of Science and Google scholar. The following keywords and MeSH terms were used: [“malaria” or “falciparum” or “vivax” or “plasmodium”] AND [“toll-like receptor 4” or “TLR-4” or “toll-4 receptor” or “toll 4 receptor” or “toll-like receptor 9” or “TLR-9” or “toll-9 receptor” or “toll 9 receptor”].

A search strategy in PubMed is provided in Additional file 2. Studies that matched the inclusion criteria were retrieved. References of the retrieved articles were manually screened to capture any additional studies.

### Selection criteria

Studies included had to meet all the following criteria:

(i) Human studies;
(ii) Case–control design of association studies:
(iii) Assessed the relationship of TLR4 (D299G, T399I) or TLR9 (T1237C, T1486C) polymorphisms (rs 187084, rs 5743836, rs 4986791) with malaria risk;
(iv) Cases had conformed to the diagnostic criteria of malaria;
(v) Genotype distributions in the cases and controls were available for estimating the odds ratio (OR) and respective 95% confidence interval (CI); and
(vi) The distribution of genotypes in the control group was consistent with Hardy–Weinberg equilibrium (HWE) [17].

When several studies used the same subjects, a publication with the largest sample size was considered.

### Data extraction

One investigator (CN) screening the studies, following four-phases study selection process and chose the eligible study. This was cross-checked by another investigator (WST). For each study included, two investigators (WST, CN) independently extracted information using a piloted data extraction sheet. Collected information were the first author name, publication year, study year, study country, number of cases/controls, age group, male%, source of controls, detection methods, polymorphism frequencies in cases and controls, ethnic group (if provided) and Hardy*–*Weinberg equilibrium (HWE) status (if not provided, HWE was calculated by this research team). The two investigators assessed the quality of eligible studies using the Newcastle–Ottawa scale (NOS) which covers three main domains (selection, exposure, comparability) in eight items [18]. The discrepancy was resolved by consensus or discussion with the third investigator (HHA). Each item was awarded 1 or 2 stars in maximum for high quality, and a final score obtained was between 0 and 9 stars. Studies with ≥ 7 stars were deemed of high quality.

### Data synthesis

The strength of the association between the TLR4/TLR9 genetic polymorphisms with the risk of malaria was assessed with OR and its 95% CI. This meta-analysis study examined the association between TLR4 (D299G, T399I) polymorphisms or TLR9 (T1237C, T1486C) and malaria risk under five genetic models. As an example for TLR9 (T1486C), allele model (C vs T), dominant model (CC + TC vs TT), recessive model (TT vs TC + TT), homozygous model (CC vs TT), and heterozygous model (TC vs TT) were used.

To investigate robustness of effect estimates, analysis was stratified by geographical region (Asian countries vs non-Asian countries) and age group. If adults’ participants in the included studies were all from Asian countries or children were all from non-Asian countries, combined subgroups were introduced (i.e. adults of Asian countries, children of non-Asian countries). This was a case for TLR9 (T1237C, T1486C) and TLR4 (D299G). If data allowed only for a subgroup analysis by parasite speciation, it was stratified into two groups *Plasmodium falciparum* and *Plasmodium vivax*. This was a case for TLR4 (D299G). The heterogeneity of the included articles was determined with *I*^*2*^ statistics. The *I*^*2*^ ≥ 50% was regarded as substantial heterogeneity, and a random effects model was used for pooling of studies. Otherwise, a fixed effect model was used [19]. For sensitivity analysis, the effect estimates were recalculated after removal of studies which had absence of HWE conformity. Funnel plot was planned to evaluate the publication bias. Due to the limited number of studies included in each SNP under specific genetic model (i.e. < 10 studies), it was not done. Data analysis was carried out with Review Manager 5.4 and R version 3.6.1.

## Results

**Fig. 1:**
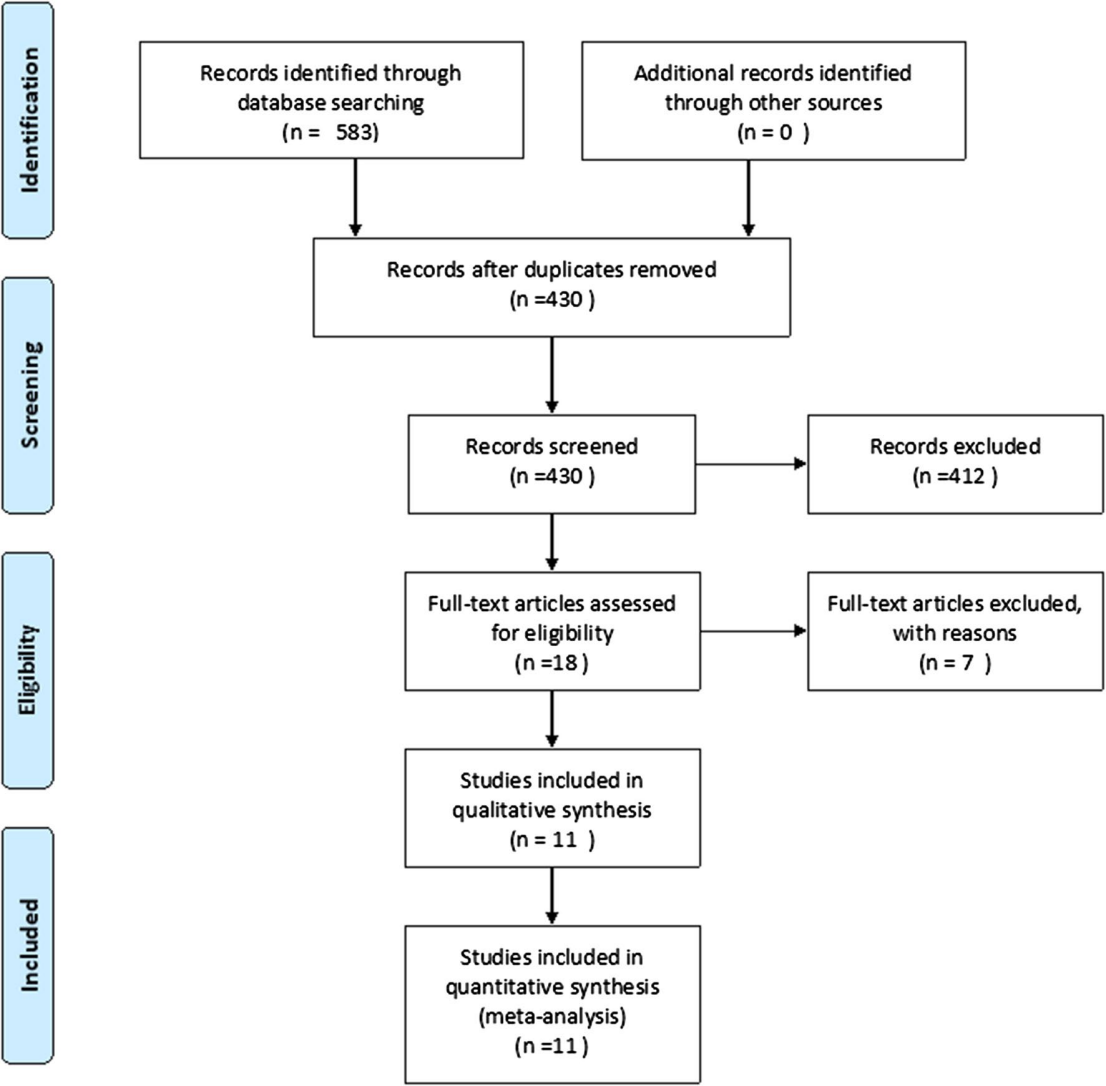
Study selection process

The study selection process is presented in Fig. 1. A total of 583 hits were retrieved from the databases in the initial search. Of these, a total of 153 duplicates were removed by checking the titles and abstracts. After further processing, 18 full-text publications were evaluated. Of these, a final of 11 studies were included [20–30]. Seven studies were excluded as they did not meet the inclusion criteria. The justification for exclusion of these studies is presented in Additional file 3.

### Study characteristics and quality assessment

**Fig. 2:**
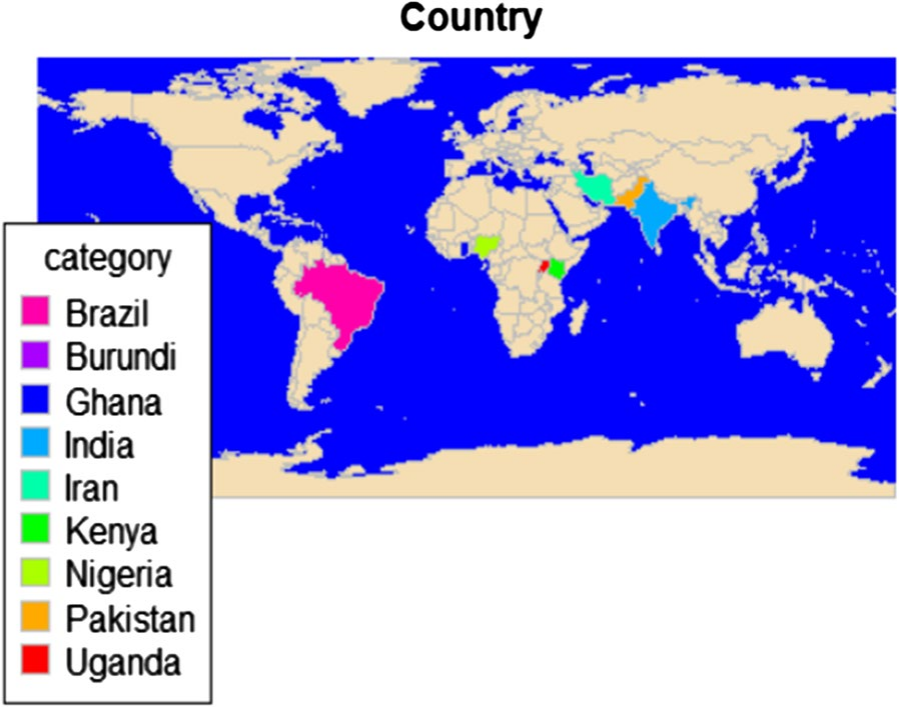
Geographic distribution of the included studies

The main characteristics of included studies are summarized in Table 1. The present meta-analysis included 11 studies incorporating 2716 cases and 2376 controls, with malaria from nine endemic countries which consist of two each in Brazil and India, and seven single studies in Burundi, Ghana, Iran, Kenya, Nigeria, Pakistan and Uganda (Fig. 2). Nine studies assessed TLR 9(T1237C), ten studies assessed TLR4 (D299G) and eight studies assessed TLR9 (T1486C), while five studies for TLR 4 (T399I). Frequency of polymorphisms are provided in Additional file 4.

**Table 1:** Characteristics of the included studies

Author, year [Ref]	SNP	Country	Setting	Age group in cases,yr	Male % in cases	Cases	Controls	Genotyping method	Consistent with HWE
Mockenhaupt, 2006 [20]	TLR4-A299G, T399I TLR9 T-1237C, T1486C	Ghana	H	Children	46.6	290	UM: 290 Healthy: 290	allele-specific PCR and PCR-RT	Yes
Leoratti, 2008 [21]	TLR4 D299G TLR9 T1237C, T1486C	Brazil	H	md 29	86	230	74	PCR–RFLP	Yes
Sam-Agudu, 2010 [22]	TLR4-A299G, T399I TLR9-T1486C, T1237C	Uganda	H	6.2 (2.4)	63.1	Cases (SM): 65	Controls (UM: 52	PCR and LDR-FMA	Yes
Zakeri, 2011 [23]	TLR4 D299G, T399I TLR9 T1486C, T1237C	Iran	HCs	md 28	65.6	P. falciparum infected: 320	Healthy control: 320	PCR–RFLP	Yes
Esposito, 2012 [24]	TLR4 A299G (rs4986790) TLR9 T1486C (rs187084), T1237C (rs5743836)	Burundi	H	Children	57.5	602	337	Taqman	Yes
Munde, 2012 [25]	TLR9 T1237C	Kenya	H	Children	47.8	SM: 138	UM: 163	TaqMan	Yes
Sawian, 2012 [26]	TLR4 A299G TLR9 T1237C, T1486C	India	H	md: 26.2 (R2-58)		SM: 136	Controls (UM: 53	PCR–RFLP	No
Kar, 2015 [27]	TLR4 D299G TLR9 T1237C, T1486C	India	H	SM: 38.0 (25.0–53.7) UM: 38.0 (24.2–55.0)	58%	200	200	PCR–RFLP	Yes (D299G) No (T1237C and T1486C)
Iwalokun, 2015 [28]	TLR4 A299G, T399I	Nigeria	H	Children	62.1	182	97	PCR–RFLP	Yes
Costa, 2017 [29]	TLR4 A299G (rs4986790), T399I (rs4986791) TLR9-T1237C (rs187084), T1486C (rs5743836)	Brazil		37 (19 ± 53)	61.5	*Pv*-325	Healthy group: 274	PCR–RFLP	Yes (A299G, T399I, T1237C) No (T1486C)
Rani, 2018 [30]	TLR4 A299G	Pakistan	H	22.7 ± 13.6	53.5	228 (SM: 89, UM: 139)	226	Allele-specific PCR	No

*H* hospital, *HC* Primary Health Centre, *HWE* Hardy*–*Weinberg equilibrium, *LDR-FMA* ligase detection reaction-fluorescent microsphere assay, *md* median, *PCR–RFLP* polymerase chain reaction-restriction fragment length polymorphism fragment length, *PCR-RT* real-time PCR, *R* range, *SM* severe malaria/complicated malaria/cerebral malaria, *SNP* single nucleotide polymorphism, *TaqMan* TaqMan allelic discrimination/SNP assay, *UM* uncomplicated malaria/mild malaria, *Yes/No* controls are/are not consistent with HWE

Publication period of the studies identified for this review spanned from 2006 to 2018. Six studies were done with adults [21, 26–30], while remaining five studies were done with children [20, 22–25]. Regarding the quality assessment, all included studies had a range of four to eight scores, and five studies (45.4%) obtained high quality score (≥ 7 stars) (Additional file 5).

### Effect of TLR 4/TLR9 on severity outcome

The association of TLR 4/TLR 9 and severity of malaria was reported in nine studies for TLR9 (T1237C) [20–27, 29], eight studies for TLR9 (T1486C) [20–24, 26, 27, 29], ten studies for TLR4 (D299G) [20–24, 26–30] and five studies for TLR4 (T399I) [20, 22, 23, 28, 29].

**Fig. 3:**
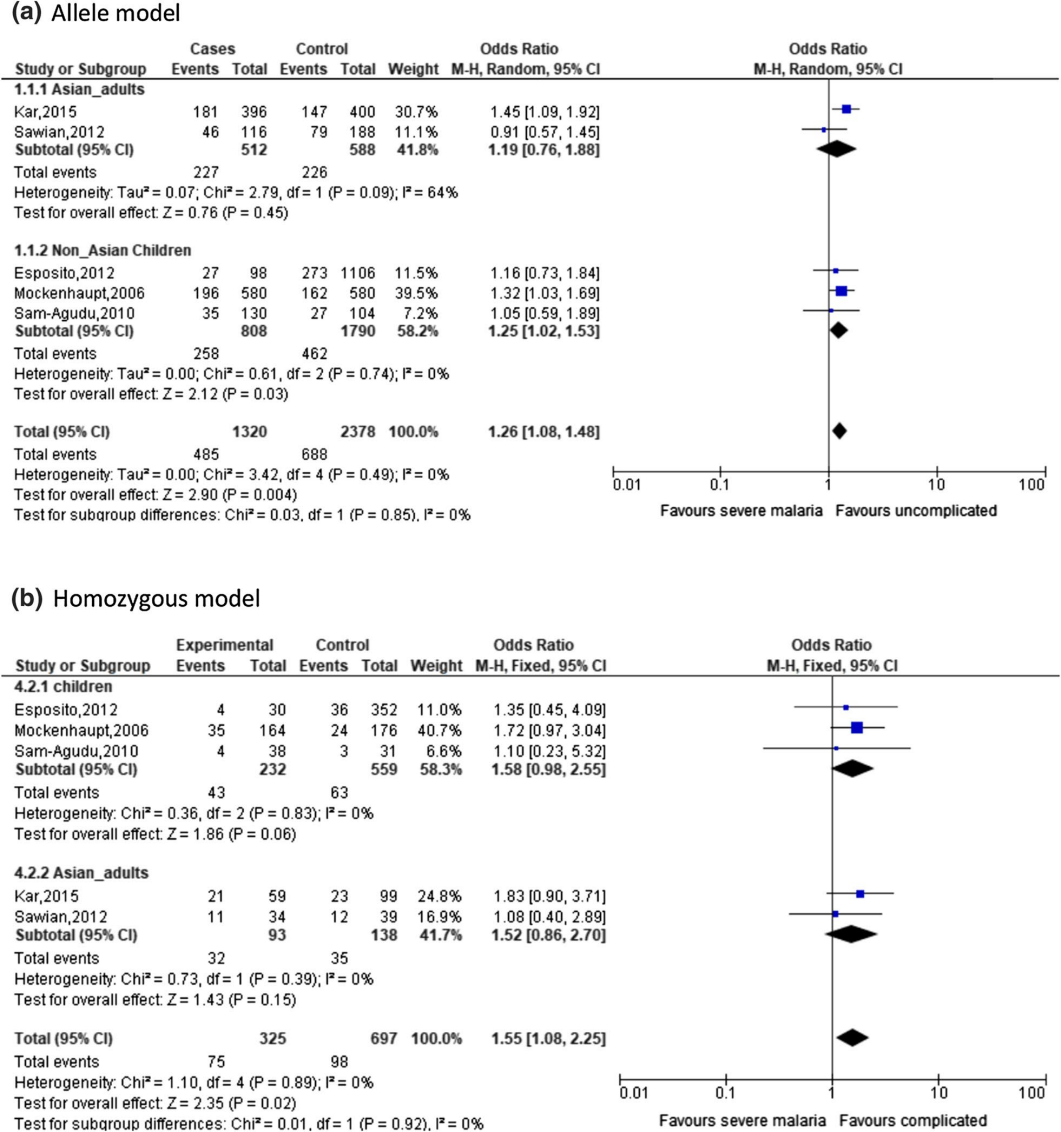
Forest plot for TLR 9 (T1486C) in severe malaria

**Fig. 4:**
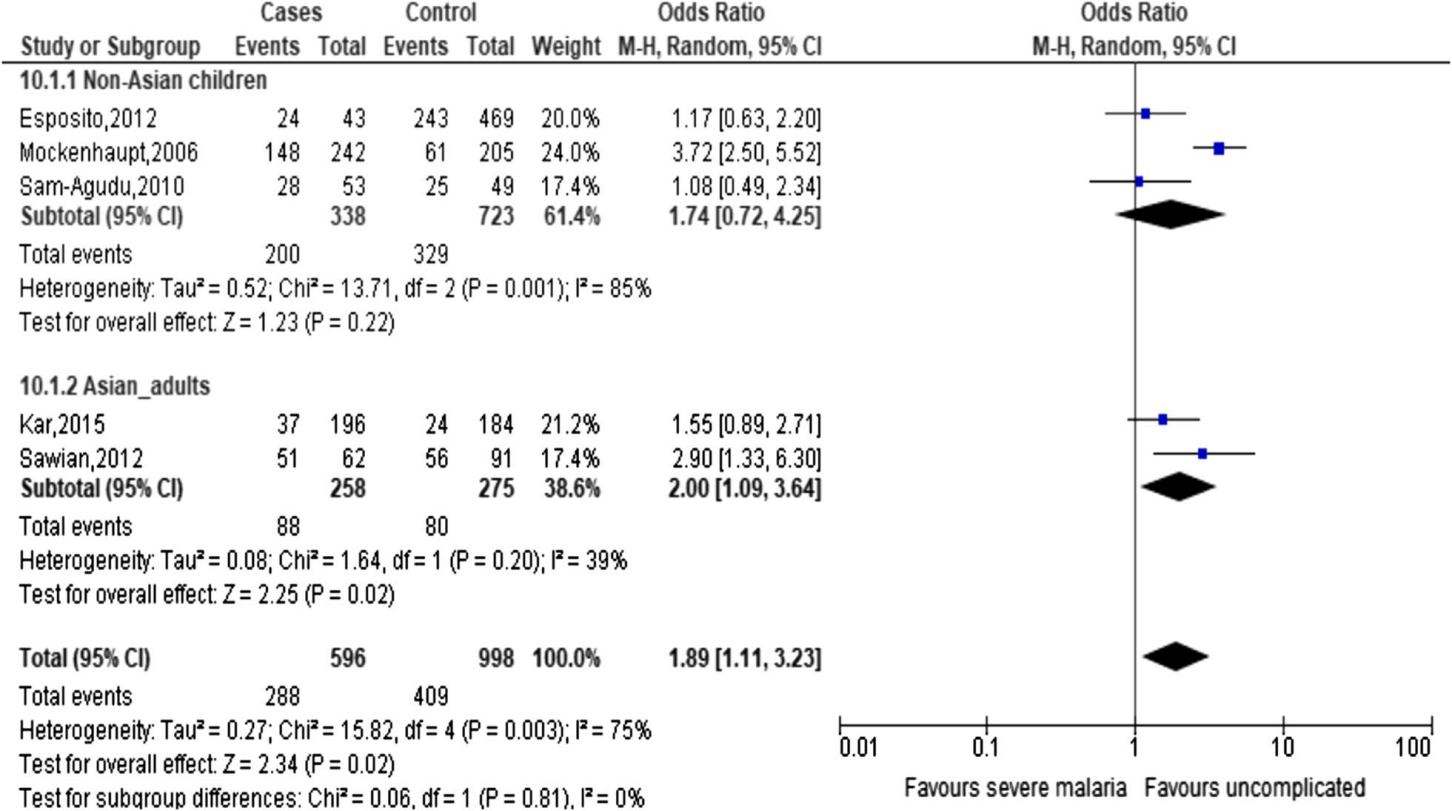
Forest plot for TLR 9 (T1237C) in severe malaria

A significant association between TLR9 (T1486C) and severity of malaria is observed in allele model (OR: 1.26, 95% CI: 1.08–1.48, *I*^2^ = 0%) or homozygous model (OR: 1.55, 95% CI: 1.08–2.28, *I*^2^ = 0%) (Fig. 3). For the remaining three genetic models (heterozygous, recessive and dominant models), none is significantly associated with the risk of severe malaria (Table 2). For other polymorphisms such as TLR9 (T1237C), a significant association with severity of malaria is observed in only heterozygous model (OR: 1.89, 95% CI: 1.11–3.22, *I*^2^ = 75%) (Fig. 4). For other polymorphisms, such as TLR 4 (D299G) and TLR4 (T399I), none of the five genetic models show significant association with severity of malaria (Table 2).

**Table 2:** Associations for the risk of severe malaria

Polymorphisms	Variants	Number of studies included	Total cases/controls	Genetic model	Effect size, OR (95%CI)	Model
TLR 9	T1486C	5	1380/2378	Allele	**1.26 (1.08–1.48)**	**F**
			660/1189	Dominant	1.29 (0.84–1.98)	R
			660/1189	Recessive	1.27 (0.9–1.79)	**F**
			325/697	Homozygous	**1.55 (1.08–2.28)**	**F**
			585/1091	Heterozygous	1.26 (0.73–2.16)	R
TLR 9	T1237C	6	1606/2720	Allele	1.01 (0.81–1.27)	R
			803/1350	Dominant	1.32 (0.86–2.03)	R
			803/1350	Recessive	0.71 (0.36–1.42)	R
			442/854	Homozygous	0.87 (0.45–1.7)	R
			722/1145	Heterozygous	1.57 (0.86–2.86)	R
TLR 4	T399I	2	202/152	Allele	1.03 (0.47–2.28)	F
			104/79	Dominant	1.05 (0.43–2.8)	F
			104/79	Recessive	0.73 (0.1–5.31)	F
			85/57	Homozygous	0.74 (0.1–5.41)	F
			102/77	Heterozygous	1.11 (0.43–2.91)	F
TLR 4	D299G	7	1463/2936	Allele	1.16 (0.93–1.44)	F
			773/1495	Dominant	1.1 (0.87–1.4)	F
			773/1495	Recessive	0.99 (0.78–1.26)	F
			625/1207	Homozygous	1.29 (0.65–2.55)	F
			759/1478	Heterozygous	1.08 (0.84–1.37)	F

*CI* confidence interval, *F* fixed-effect model, *OR* odds ratio, *R* random-effects model

Significant result is in bold

Due to availability of data, stratified analysis on age group (adults and children) and geographical region (Asian countries and non-Asian countries) were done. For TLR9 (T1486C), the adult group of Asian countries is significantly associated with severity of malaria in allele model (OR: 1.25, 95% CI: 1.02–1.53, *I*^2^ = 0%). For TLR9 (T1237C), the adult group of Asian countries is also significantly associated with severity of malaria in heterozygous model (OR: 2.00, 95% CI: 1.09–3.64, *I*^2^ = 39%) (Fig. 4).

Due to the presence of studies which are not consistent with HWE, a sensitivity analysis was done by recalculation with studies consistent with HWE. After removal of two studies which are not consistent with HWE [26, 27] in TLR 9 (T1486C) (i.e. two studies on children), a significant association is only with children of non-Asian countries in severe malaria under allele model (OR: 1.26, 95% CI: 1.08–1.53 *I*^*2*^ = 0%) (Fig. 3). Of note is an absence of heterogeneity. After removal of the same two studies which are not consistent with HWE [26, 27], TLR 9 (T1237C) is no longer significantly associated with severe malaria under heterozygous model (OR: 1.74, 95% CI: 0.72–4.25 *I*^*2*^ = 85%) (Fig. 4). This implies that there is an impact of HWE status on the effect estimates.

### Effect of TLR 4/TLR9 on susceptibility of malaria

There is no significant association between TLR9 (T1486C) and susceptibility of malaria in any five genetic models in any subgroups (Table 3). However, on subgroup analysis, TLR9 (T1237C) is significantly associated with children group of non-Asian countries under recessive model (OR: 2.21, 95% CI: 1.06–4.57, *I*^2^ = 85%). and homozygous model (OR: 1.49, 95% CI: 1.09–2.0 *I*^2^ = 0%). Of note, there are only two studies included in this subgroup and a wide 95%CI in recessive model (Additional file 6).

**Table 3:** Associations for the risk of susceptibility to malaria

Polymorphisms	Variants	Number of studies included	Cases/controls	Genetic model	Effect size, OR (95% CI)	Model
TLR 9	T1486C	5	3472/2610	Allele	1.03 (0.91–1.16)	F
			1740/1166	Dominant	1.07 (0.78–1.47)	F
			1740/1166	Recessive	0.93 (0.68–1.27)	F
			801/533	Homozygous	0.85 (0.5–1.43)	R
			1566/1018	Heterozygous	1.17 (0.78–1.78)	R
TLR 9	T1237C	5	2867/2217	Allele	0.97 (0.84–1.12)	F
			1735/1387	Dominant	0.87 (0.52–1.45)	R
			1735/1387	Recessive	1.53 (0.86–2.75)	R
			1225/859	Homozygous	1.28 (0.97–1.68)	R
			1537/1173	Heterozygous	0.82(0.49–1.4)	R
TLR 4	T399I	4	1716/1400	Allele	0.95 (0.69–1.31)	F
			861/707	Dominant	0.99 (0.71–1.39)	F
			861/707	Recessive	1.03 (0.73–1.46)	F
			776/634	Homozygous	0.54 (0.17–1.69)	F
			826/700	Heterozygous	1.13 (0.8–1.6)	F
TLR 4	D299G	7	3776/2847	Allele	1.05 (0.86–1.29)	F
			1959/1576	Dominant	1.11 (0.9–1.36)	F
			684/1356	Recessive	0.83 (0.66–1.05)	F
			545/1092	Homozygous	0.95 (0.51–1.79)	F
			672/1341	Heterozygous	1.08 (0.87–1.34)	F

*F* fixed effect model, *R* random effects model

### Subgroup analysis and publication bias

The remaining polymorphisms of TLR4 (D299G, T399I) showed no significant association with the susceptibility of malaria or severity of malaria in any subgroup analysis. Only two studies provided data on *P. vivax* [29, 30]. Hence, it was limited to do subgroup analysis with speciation. To investigate funnel plot asymmetry, there should be at least 10 studies included as described in in the *Cochrane Handbook for Systematic Reviews of Interventions* (Chapter 13.3.5.4) [31]. As there was limited number of studies included in each SNP under specific genetic model (Tables 2, 3), it was not done.

## Discussion

The current meta-analysis study has systematically evaluated the role of TLR4/9 in severity of malaria or susceptibility to malaria risk in people living in endemic countries. This study is a comprehensive meta-analysis covering four available TLR polymorphisms extracted from 11 studies.

The summary of findings is as follows:

- TLR 9 (T1486C) showed a significant role in severity of malaria under allele model and homozygous genetic models. These were in the absence of within-study heterogeneity. A sensitivity analysis showed the stability of the estimates.
- TLR 9 (T1486C) showed a significant role in susceptibility of malaria only in the subgroup of children from non-Asian countries under allele model.
- TLR9 (T1237C) and severity of malaria was significantly associated in the adult of Asian countries under heterogeneous model. This polymorphism was significantly associated with susceptibility of malaria in the children group of non-Asian countries under recessive and homogenous genetic models
- The reaming TLR polymorphisms showed no significant association with either severity of malaria or susceptibility of malaria under any genetic models or in any subgroups.

TLR ligand recognition and adaptor specific result in the upregulation of specific pro-inflammatory cytokines and chemokines for pathogen control and clearance; however, excessive inflammation is extremely harmful or even fatal to the hosts [32]. Hence, the role of TLR is complicated as reported in this current study and limited to show associations in TLR9 (T 1486 C, T1237C). The presence of single nucleotide polymorphisms in an individual can be considered as one of the important determinants, which regulate the development of an infection [33]. Based on the current findings, polymorphism T1486C may be considered as the most influential TLR9. An animal model study reported that CD4CD25 regulatory T cells (Treg) activation by dendritic cells (DCs) stimulated via TLR9 [34]. Studies reported that haemozoin, a parasite ligand for TLR9, is a possible candidate ligand and it is abundant in parasitized RBCs, and it does not induce IFN-production upon stimulation of DCs [35]. Another study revealed that TLR9 is expressed predominantly by plasmacytoid DC, which are reported to be involved in Treg induction [36]. A role in susceptibility as well as severity risk for may be depending on specific allele. For instance, a study revealed that individuals with T allele showed resistance to pulmonary TB, while susceptibility was observed in the individuals having C allele [37]. To certain extent, this may also apply to malaria in the current study.

### Study limitations

Number of studies included for each gene polymorphisms are limited and this might contribute to type II statistical error. Due to limited data, subgroup was done with a few factors. There might be other factors that were not included in the primary studies. Despite many studies have demonstrated that the pathology of malaria is immune-mediated, non-immunological factors are also believed to play a role in the disease severity. For instance, physiological maturity, such as age, independent of malaria exposure and acquired immunity, is a significant modifier of susceptibility [38] and it is possible that there are studies that reported physiological age limits below which severe malaria presents as anaemia and above which it presents [2].

Nevertheless, the analysis of genetic factor(s) such as TLR4/9 in the present study may serve as a useful approach in identifying the patients who are at an increased risk for a malaria infection or for poor disease progression (i.e. severe malaria), leading to more aggressive treatment intervention. Several lines of evidence had reported in the importance of targeting TLRs for the prevention and treatment of several inflammatory diseases, including cancer, rheumatoid arthritis and inflammatory bowel disease [39]. TLR-targeted therapeutic intervention may usher a hope for treating the pathogenic infections such as malaria in this study.

## Conclusions

The findings suggest that TLR 9 (T1486C and T123C) seems to influence the progression of malaria, under certain genetic models and in specific age group of people from specific geographical region. TLR 9 (T1237C) also plays a role in susceptibility to malaria under certain genetic models in the children group. To substantiate these, future well designed studies with larger samples across endemic countries are recommended.

## Supplementary Information

**Additional file 1**: PRISMA checklist.

**Additional file 2**: Search strategy in PubMed.

**Additional file 3**: Excluded studies.

**Additional file 4**: Frequency of genetic polymorphisms.

**Additional file 5**: Assessment of study quality through Nos checklist.

**Additional file 6**: Forest plot for TLR 9 (T1237C) in susceptibility to malaria.

## Data Availability

All data generated or analysed during this study are included in this article and its supplementary information files.

## Abbreviations

CI: Confidence interval (CI)
DAMPs: Damage-associated molecular patterns
DC: Dendritic cells
HWE: Hardy–Weinberg equilibrium
NOS: Newcastle–Ottawa scale
OR: Odds ratio
PAMPs: Pathogen-associated molecular patterns
PRR: Pattern recognition receptors
TLRs: Toll like receptors
Treg: Regulatory T cells
