# Supplementary material for: Toll-like receptor 9 and 4 gene polymorphisms in susceptibility and severity of malaria: a meta-analysis of genetic association studies
Source: Malar J. 2021 Jul 3;20:302. doi: 10.1186/s12936-021-03836-6 (PMC8255014; doi:10.1186/s12936-021-03836-6)
Supplement: Supplementary file 2 — Additional file 2: Search strategy in PubMed. [file 12936_2021_3836_MOESM2_ESM.doc]

**Additional File 2. Search Strategy in PubMed**

((("toll-like receptor 4"[MeSH Terms] OR "toll-like receptor 4"[All Fields] OR "toll like receptor 4"[All Fields]) OR ("toll-like receptor 4"[MeSH Terms] OR "toll-like receptor 4"[All Fields] OR "toll like receptor 4"[All Fields])) AND ("polymorphism, genetic"[MeSH Terms] OR ("polymorphism"[All Fields] AND "genetic"[All Fields]) OR "genetic polymorphism"[All Fields] OR "polymorphism"[All Fields])) AND ("malaria"[MeSH Terms] OR "malaria"[All Fields])
