# Supplementary material for: Toll-like receptor 9 and 4 gene polymorphisms in susceptibility and severity of malaria: a meta-analysis of genetic association studies
Source: Malar J. 2021 Jul 3;20:302. doi: 10.1186/s12936-021-03836-6 (PMC8255014; doi:10.1186/s12936-021-03836-6)
Supplement: Supplementary file 3 — Additional file 3: Excluded studies. [file 12936_2021_3836_MOESM3_ESM.doc]

Additional File 3 Excluded studies

| First author, year | Main reason | Citation |
| --- | --- | --- |
| Greene 2009 | No comparators for susceptibility/severity, all are asymptomatic cases | Greene, J.A., Moormann, A.M., Vulule, J. et al. Toll-like receptor polymorphisms in malaria-endemic populations. Malar J 2009; **8,** 50 |
| Basu 2010 | No comparators | Basu M, Maji AK, Chakraborty A, et al. Genetic association of Toll-like-receptor 4 and tumor necrosis factor-alpha polymorphisms with *Plasmodium falciparum* blood infection levels. Infect Genet Evol. 2010;10(5):686-96. |
| Iwolokan  2017 | No separate data for TLR4 (299) & TLR 4 (399) | Iwalokun BA, Iwalokun SO, Udoh BE, Balogun M. Assessment of co-segregated TLR4 genotypes among Nigerian children with asymptomatic and clinical malaria. Asian Pac J Trop Biomed. 2017; 7: 96–102 |
| Mockenhaupt, 2006 | Pregnant women | Mockenhaupt FP, Hamann L, von Gaertner C et al. Common polymorphisms of toll-like receptors 4 and 9 are associated with the clinical manifestation of malaria during pregnancy. J Infect Dis. 2006;194(2):184-8. |
| Long, 2014 | Not an association study in malaria | Long H, O'Connor BP, Zemans RL et al. The Toll-like receptor 4 polymorphism Asp299Gly but not Thr399Ile influences TLR4 signaling and function. PLoS One. 2014;9(4):e93550. |
| Barboza, 2018 | Not an association study | Barboza R, Lima FA, Reis AS, et al. TLR4-mediated placental pathology and pregnancy outcome in experimental malaria [published correction appears in Sci Rep. 2018 Mar 6;8(1):4275]. Sci Rep. 2017;7(1):8623. |
| Apinjo,2013 | Frequency data are not provided. | Apinjoh TO, Anchang-Kimbi JK, Njua-Yafi C et al. Association of cytokine and toll-like receptor gene polymorphisms with Severe Malaria in three regions of Cameroon. PLoS One 2013; 8(11): e81071. |
