# Supplementary material for: Toll-like receptor 9 and 4 gene polymorphisms in susceptibility and severity of malaria: a meta-analysis of genetic association studies
Source: Malar J. 2021 Jul 3;20:302. doi: 10.1186/s12936-021-03836-6 (PMC8255014; doi:10.1186/s12936-021-03836-6)
Supplement: Supplementary file 4 — Additional file 4: Frequency of genetic polymorphisms. [file 12936_2021_3836_MOESM4_ESM.doc]

Additional File 4

TLR 4 (D299G)

| **study** | **yr** | **country** | **age group** | species | **DD** | **DG** | **GG** | **DD** | **DG** | **GG** | D | G | D | G | comparison |
| --- | --- | --- | --- | --- | --- | --- | --- | --- | --- | --- | --- | --- | --- | --- | --- |
| **Sawian** | **2012** | **India** | **adults** | Pf only | **50** | **9** | **3** | **78** | **9** | **4** | 109 | 15 | 22 | 222 | SCM vs UM |
| **Kar** | **2015** | **India** | **adults** | Pf only | **141** | **52** | **6** | **146** | **49** | **3** | 334 | 64 | 341 | 55 |  |
| **Sam-Agudu** | **2010** | **Uganda** | **children** | Pf only | **57** | **8** | **0** | **47** | **5** | **0** | 122 | 8 | 99 | 5 |  |
| **Esposito** | **2012** | **Burundi** | **children** | Pf only | **47** | **7** | **0** | **481** | **70** | **2** | 101 | 7 | 1032 | 74 |  |
| **Mockenhaupt** | **2006** | **Ghana** | **children** | Pf only | **220** | **65** | **5** | **224** | **64** | **2** | 505 | 75 | 512 | 68 |  |
| **Iwolokan** | **2015** | **Nigeria** | **adults** | Pf only | **12** | **4** | **1** | **152** | **16** | **2** | 28 | 6 | 320 | 20 |  |
| **Rani** | **2018** | **Iran** | **adults** | Pv mainly | **78** | **9** | **2** | **114** | **21** | **4** | 82 | 7 | 208 | 18 |  |
| Sawian | 2012 | India | adults | Pf only | 78 | 9 | 4 | 56 | 9 | 3 | 165 | 17 | 121 | 15 | Infected vs uninfected |
| Esposito | 2012 | Burundi | children | Pf only | 528 | 72 | 2 | 300 | 36 | 1 | 1128 | 76 | 636 | 38 |  |
| Mockenhaupt | 2006 | Ghana | children | Pf only | 224 | 64 | 2 | 239 | 47 | 4 | 512 | 68 | 525 | 55 |  |
| Zakeri | 2011 | Iran | adults | Pf only | 287 | 33 | 5 | 276 | 39 | 0 | 607 | 43 | 591 | 39 |  |
| Costa | 2017 | Brazil | adults | pv | 312 | 13 | 0 | 260 | 14 | 0 | 637 | 13 | 534 | 14 |  |
| Leoratti | 2008 | Brazil | adults | pf only | 184 | 16 | 2 | 40 | 6 | 0 | 384 | 20 | 86 | 6 |  |
| Rani | 2018 | Iran | adults | pv | 114 | 21 | 4 | 196 | 26 | 14 | 125 | 14 | 208 | 18 |  |

TLR4(T 399I)

| **study** | **yr** | **country** | **age group** | **species** | **AA** | **AG** | **GG** | **AA** | **AG** | **GG** | **Comparison** |
| --- | --- | --- | --- | --- | --- | --- | --- | --- | --- | --- | --- |
| Sam-Agudu | 2010 | Uganda | children | pf only | 65 | 2 | 1 | 53 | 1 | 1 | **SCM vs UM** |
| Mockenhaupt | 2006 | Ghana | children | Pf only | 18 | 17 | 1 | 12 | 11 | 1 | **SCM vs UM** |
| Iwolokan | 2015 | Nigeria | adults | Pf only | 177 | 8 | 2 | 88 | 0 | 4 | **infected vs non** |
| Costa | 2017 | Brazil | adults | pv | 310 | 16 | 1 | 262 | 14 | 1 | **infected vs non** |
| Zakeri | 2011 | Iran | adults | Pf only | 272 | 50 | 1 | 271 | 51 | 1 | **infected vs non** |
| Mockenhaupt | 2006a | Ghana | children | Pf only | 12 | 11 | 1 | 7 | 7 | 0 | **infected vs non** |

TLR9 (T1237C)

| **study** | **yr** | **country** | **age group** | **species** | **TT** | **TC** | **CC** | **TT1** | **TC1** | **CC1** | **Comparison** |
| --- | --- | --- | --- | --- | --- | --- | --- | --- | --- | --- | --- |
| Munde | 2012 | Kenya | children | pf only | 63 | 63 | 12 | 60 | 87 | 16 | **SCM vs UM** |
| Mockenhaupt | 2006 | Ghana | children | pf only | 94 | 148 | 48 | 144 | 61 | 85 | **SCM vs UM** |
| Sawian | 2012 | India | adults | pf only | 11 | 51 | 1 | 35 | 56 | 1 | **SCM vs UM** |
| Sam-Agudu | 2010 | Uganda | children | pf only | 25 | 28 | 12 | 24 | 25 | 3 | **SCM vs UM** |
| Kar | 2015 | India | adults | pf only | 159 | 37 | 2 | 160 | 24 | 16 | **SCM vs UM** |
| Esposito | 2012 | Burundi | children | pf only | 19 | 24 | 6 | 226 | 243 | 84 | **SCM vs UM** |
| Leoratti | 2008 | Brazil | adults | pf only | 140 | 50 | 8 | 32 | 12 | 2 | **infected vs non** |
| Mockenhaupt | 2006a | Ghana | children | pf only | 144 | 61 | 85 | 106 | 148 | 46 | **infected vs non** |
| Costa | 2017 | Brazil | adults | Pv | 222 | 93 | 10 | 192 | 76 | 6 | **infected vs non** |
| Esposito | 2012 | Burundi | children | pf only | 245 | 267 | 90 | 155 | 147 | 35 | **infected vs non** |
| Zakeri | 2011 | Iran | adults | Pf only | 276 | 39 | 5 | 270 | 35 | 15 | **infected vs non** |

TLR 9(T1486C)

| **study** | **yr** | **country** | **age group** | **species** | **TT** | **TC** | **CC** | **TT** | **TC** | **CC** | **Group** |
| --- | --- | --- | --- | --- | --- | --- | --- | --- | --- | --- | --- |
| Leoratti | 2008 | Brazil | adults | pf only | 70 | 98 | 31 | 18 | 23 | 4 | infected vs non |
| Mockenhaupt | 2006 | Ghana | children | pf only | 138 | 114 | 24 | 162 | 134 | 28 | infected vs non |
| Costa | 2017 | Brazil | adults | Pv | 98 | 183 | 44 | 65 | 153 | 56 | infected vs non |
| Esposito | 2012 | Burundi | children | pf only | 342 | 220 | 40 | 197 | 113 | 27 | infected vs non |
| Zakeri | 2011 | Iran | adults | pf only | 142 | 147 | 35 | 130 | 157 | 33 | infected vs non |
| Mockenhaupt | 2006a | Ghana | children | pf only | 161 | 126 | 35 | 138 | 114 | 24 | SCM vs UM |
| Sawian | 2012 | India | adults | Pf only | 23 | 24 | 11 | 27 | 55 | 12 | SCM vs UM |
| Sam-Agudu | 2010 | Uganda | children | pf only | 34 | 27 | 4 | 28 | 21 | 3 | SCM vs UM |
| Kar | 2015 | India | adults | pf only | 38 | 139 | 21 | 76 | 101 | 23 | SCM vs UM |
| Esposito | 2012 | Burundi | children | pf only | 26 | 19 | 4 | 316 | 201 | 36 | **SCM vs UM** |

SCM: severe/ complicated malaria; UM: uncomplicated/mild malaria; Infected vs non-infected = Uncomplicated/ mild malaria vs healthy control/uninfected with malaria
