# Supplementary material for: Toll-like receptor 9 and 4 gene polymorphisms in susceptibility and severity of malaria: a meta-analysis of genetic association studies
Source: Malar J. 2021 Jul 3;20:302. doi: 10.1186/s12936-021-03836-6 (PMC8255014; doi:10.1186/s12936-021-03836-6)
Supplement: Supplementary file 5 — Additional file 5: Assessment of study quality through Nos checklist. [file 12936_2021_3836_MOESM5_ESM.doc]

**Additional File 5: Assessment of study quality through NOS** checklist

| Item description | Mockenhaupt, 2006 [20] | Leoratti, 2008  [21] | Sam-Agudu, 2010  [22] | Zakeri, 2011  [23] | Esposito, 2012  [ 24] | Munde, 2012  [25] | Sawian, 2012  [26] | Kar, 2015  [27] | Iwalokun, 2015  [28] | Costa, 2017  [29] | Rani, 2018  [30] |
| --- | --- | --- | --- | --- | --- | --- | --- | --- | --- | --- | --- |
| Selection |  |  |  |  |  |  |  |  |  |  |  |
| **1) Is the case definition adequate?** a) yes, with independent validation * b) yes, eg record linkage or based on self reports c) no description | **1** | **1** | **1** | **1** | **1** | **1** | **1** | **1** | **1** | **1** | **1** |
| **2) Representativeness of the cases** a) consecutive or obviously representative series of cases * b) potential for selection biases or not stated | **1** | **1** | **1** | **1** | **1** | **1** | **1** | **1** | **1** | **1** | **1** |
| **3) Selection of Controls** a) community controls * b) hospital controls c) no description | **1** | 1 | 0 | 1 | 0 | 0 | 1 | 0 | 0 | 1 | 1 |
| **4) Definition of Controls** a) no history of disease (endpoint) * b) no description of source | **1** | 0 | 0 | **1** | **1** | 0 | **1** | 0 | 0 | **1** | 1 |
| Comparability |  |  |  |  |  |  |  |  |  |  |  |
| **1) Comparability of cases and controls on the basis of the design or analysis (study adjusts for age*, sex)** a) study controls for _______________ (Select the most important factor.) * b) study controls for any additional factor * (This criteria could be modified to indicate specific  control for a second important factor.) | **2** | **0** | **0** | **0** | **2** | **2** | **0** | **2** | **2** | **1** | **2** |
| Exposure |  |  |  |  |  |  |  |  |  |  |  |
| **1) Ascertainment of exposure** a) secure record (eg surgical records) * b) structured interview where blind to case/control status * c) interview not blinded to case/control status d) written self report or medical record only e) no description | **1** | **1** | **1** | **1** | **1** | **1** | **1** | **1** | **1** | **1** | **1** |
| **2) Same method of ascertainment for cases and controls** a) yes * b) no | **1** | **1** | **1** | **1** | **1** | **1** | **1** | **1** | **1** | **1** | **1** |
| **3) Non-Response rate** a) same rate for both groups * b) non respondents described c) rate different and no designation | **0** | **0** | **0** | **0** | **0** | **0** | **0** | **0** | **0** | **0** | **0** |
| Total Score | 8 | 5 | 5 | 6 | 7 | 6 | 6 | 7 | 6 | 7 | 8 |
