# Supplementary material for: Toll-like receptor 9 and 4 gene polymorphisms in susceptibility and severity of malaria: a meta-analysis of genetic association studies
Source: Malar J. 2021 Jul 3;20:302. doi: 10.1186/s12936-021-03836-6 (PMC8255014; doi:10.1186/s12936-021-03836-6)

**Additional File 6. Forest plot for TLR 9 (T1237C) in susceptibility to malaria**

1. Recessive model


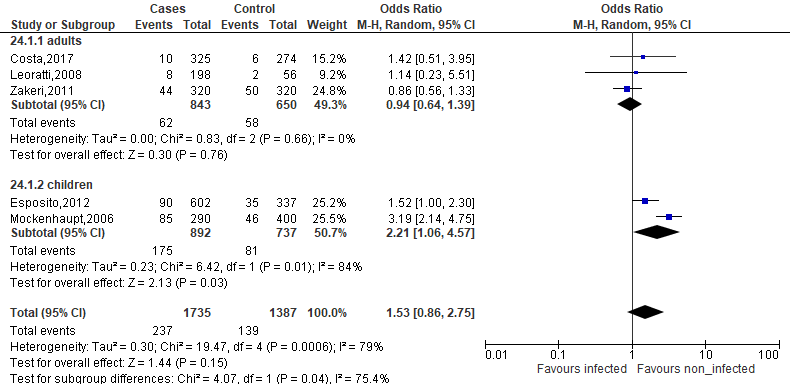


1. homozygous model


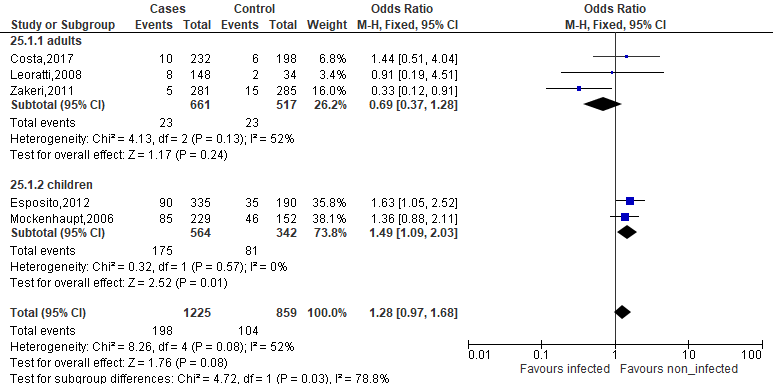

Supplement: Supplementary file 6 — Additional file 6: Forest plot for TLR 9 (T1237C) in susceptibility to malaria. [file 12936_2021_3836_MOESM6_ESM.doc]
